# Mergers and integrated care: the Quebec experience

**DOI:** 10.5334/ijic.1140

**Published:** 2013-02-22

**Authors:** Louis Demers

**Affiliations:** École nationale d’administration publique, 555, boulevard Charest Est, Québec (Québec), G1K 9E5, Canada

**Keywords:** integration, merger, health and social services, integrated care, Canada

## Abstract

As a researcher, I have studied the efforts to increase the integration of health and social services in Quebec, as well as the mergers in the Quebec healthcare system. These mergers have often been presented as a necessary transition to break down the silos that compartmentalize the services dispensed by various organisations. A review of the studies about mergers and integrated care projects in the Quebec healthcare system, since its inception, show that mergers cannot facilitate integrated care unless they are desired and represent for all of the actors involved an appropriate way to deal with service organisation problems. Otherwise, mergers impede integrated care by creating increased bureaucratisation and standardisation and by triggering conflicts and mistrust among the staff of the merged organisations. It is then preferable to let local actors select the most appropriate organisational integration model for their specific context and offer them resources and incentives to cooperate.

## Mergers and integrated care: the Quebec experience

As a researcher, I have studied the efforts to increase the integration of health and social services in Quebec, as well as the mergers that have taken place in the Quebec healthcare system. As elsewhere in developed countries, these mergers have often been presented as a necessary transition to break down the silos that compartmentalize the services dispensed by various organisations. Yet the Quebec experiences with service integration that have proven effective have been the result of interorganisational collaboration or, one might say, a network organisation [1–3]. If mergers are not necessary to increase integrated care, are they more useful or more detrimental to this form of care?

In 2002, two colleagues and I asked the question directly: “Is integrated care conditional on institutional mergers?” [4, p. 74]. We maintained at the time that the relevance of organisational integration mechanisms depends on their effect on the collaboration of practitioners, that is to say on the willingness and ability of the practitioners to work together on a service integration project. We spoke in particular about the importance of informal organisational modes, such as communities of practice, to produce integrated care. A merger would then only be useful if it made sense for the practitioners and supported the collaborative links within their communities.

It is in this light that I will present the results of a series of empirical studies carried out in Quebec on mergers of public institutions and put them in an international perspective. Examination of these mergers will make it possible to verify whether or not the interpretation that we proposed in 2002 still holds true.

## Forty years of mergers: is big beautiful?

The creation of Quebec’s modern healthcare system dates to the 1970s. At that time, the system had close to 1000 public institutions. Forty years later, fewer than 200 remain [5]. This dramatic reduction is the result of waves of mergers encouraged or imposed by the government and of some initiatives by the management of neighbouring institutions.

Bégin [6] and Bégin and Labelle [7] studied two series of mergers that were imposed on small institutions located in outlying regions. In the early 1970s, nine existing general hospitals were each linked with their local community services centre, a new organisation devoted to preventive and primary care services. Four years after their creation, these mergers have all proven to be “highly unstable and ineffective” [8, p. 36]. In 1977, the Department of Social Affairs created eight health centres, which also comprised a small general hospital and a local community services centre. Ten years after their creation, these health centres are administratively integrated, but their services remain compartmentalized [7].

In the two series of mergers, the authors explain the poor collaboration between the hospital staff and the local community services centre staff as a result of the significant differences in their values and intervention methods. In the first group of mergers, the physicians and employees of the hospital and those of the local community services centre remain two sub-cultures, two distinct coalitions. This divergence was accentuated by the obligation imposed on the parties to integrate with one another, which no one wanted. This top-down implementation strategy fed a climate of animosity in the second group of mergers and did not elicit the commitment of the physicians and staff of the health centres.

At the end of the 1990s, I co-led a study on the relations between institutional integration and integrated care in one region of Quebec [9]. My co-researchers and I observed that mergers could considerably impede service integration projects when they were feared by either of the targeted institutions. The threat of forced merger triggered reactions of mistrust and led to the stagnation of service integration projects for the elderly. In contrast, some mergers were negotiated voluntarily. They linked organisations of comparable size, which avoided the risk of the smaller organisation being absorbed by a more powerful organisation, usually the hospital. When the possibility of a merger was evacuated, managers and stakeholders willingly collaborated on service integration projects, with full trust and respect for one another’s mission.

In the 1980s and 1990s, several horizontal mergers, that is to say linking institutions pursuing the same mission, took place. Most often imposed by government authorities in order to cut costs and streamline services, these mergers provoked vigorous opposition and took years to be negotiated. Whether they involved small general hospitals located in the regions [10] or university hospitals in large urban centres [11], these mergers consumed considerable time and energy without leading to notable improvements in the organisation of services, at least after the first few years. Even the voluntary mergers carried out at that time required years of negotiations before they were effectively completed [8]. Mergers of hospitals in different national contexts led to similar, disappointing results [12–15].

In 2004, a widespread reform divided Quebec into 95 sub-regions, each one endowed with a new organisation: the health and social service centre. These centres are comprised of all the local community services centres and the residential and long-term care centres on their territory plus, for the large majority of them, a general hospital. Each centre is responsible for coordinating a local network of services that includes specialized institutions, community organisations and physicians’ offices. The primary goal of the reform was to ensure accessibility and continuity of care through an integrated provision of services [5].

Three research studies, one of which is ongoing, conducted in 14 different health and social service centres between four and six years after their creation shed light on the effect of the mergers on the conditions for achieving a greater integration of services [16–17]. The CSSS studied are quite varied as to the milieu in which they are located (urban, rural), the size and number of organisations they integrate and whether they are the result of a mandatory or voluntary merger. In the three studies, mergers are seen more as slowing than accelerating the changes sought by the reform.

On the one hand, the first years of the merged health and social service centres were devoted to introducing the new administrative structure and to filling management positions. It was not until this ‘organisational project’ was completed that the managers attended to the ‘clinical project’ that aimed to plan the service networks for specific clienteles.

Secondly, several centres became large organisations, with a few thousand employees working in several service points. For the sake of equity, budgets and rules were ‘harmonized’ across the centre, which meant that well established ways of doing things had to be abandoned, a phenomenon increased by the fact that several managers were new to their positions. In general, the frontline workers perceive little change in their practice and little progress in service integration. For them, the mergers are mainly synonymous with administrative red tape and distancing from decision-making processes. The fact of including a hospital in a health and social service centre complicates the organisational and clinical integration processes as they must standardise very different ways of doing things.

The mergers that seem to give the best results are those that arise from a voluntary choice or that create an organisation that remains on a human scale. These conditions facilitate mutual adjustment and the sharing of common norms to forge collaborative links within the health and social service centre and between it and its partners [4, 18].

Empirical studies in Sweden [15] and the UK [19] show that conflict of values, mistrust and opposition from professionals and other stakeholders are chronic features of top-down forced mergers. More generally, in these countries as in the US, the outcomes of mergers fell short of expectations. To a large extent, neither economy of scale or scope [15, 20], nor better integrated care [19–22] has been observed. In some cases, organisational integration has impeded integrated care [19, 21]. In short, big is seldom better [12, 14] and imposed decisions rarely bring cooperation among potential partners.

## Conclusion—key lessons and insights

Policy-makers and health care organisations executives often believe that organisational integration leads to, or even equates with, integrated care [23, 24]. This assumption doesn’t hold true in practice. Healthcare and social services present a high degree of complexity, the reason they are dispensed by professional practitioners; the latter must use their judgement and knowledge, sometimes tacit, to properly do their work [23]. The use of mergers to make these practitioners collaborate reflects a mechanistic conception of professional organisations [25] that ignores the informal ties that structure collaboration in the field. This is the argument that we referred to at the beginning of this article and that our analysis supports. Based on this analysis and the studies that I have conducted on service integration projects, I propose below a few avenues to promote integrated care [26].

Mergers cannot facilitate integrated care unless they are desired and unless they represent for all of the actors involved an appropriate way to deal with service organisation problems. Otherwise, they impede integrated care by creating increased bureaucratisation and standardisation and by triggering conflicts and mistrust among the staff of the merged organisations.

Rather than imposing mergers, it is preferable to offer local actors the possibility to choose the most appropriate organisational integration model for their specific context. Health system authorities should make it clear that integrated care is a priority, support changes in practices by allocating sufficient resources to allow managers and other actors to forge collaborative ties, let them adopt a model of integrated care adapted to their local conditions, introduce financial and normative incentives to collaboration, and make available best practices to stimulate emulation.

## From the author

Louis Demers is a professor at the École nationale d’administration publique, in Quebec City, Canada. He has been interested for several years in innovation in the organisation of health and social services and in the public policies of this sector. His current research focuses mainly on services for the frail elderly.
